# Polyploidy in Fruit Tree Crops of the Genus *Annona* (Annonaceae)

**DOI:** 10.3389/fpls.2019.00099

**Published:** 2019-02-11

**Authors:** Carolina Martin, Maria. A. Viruel, Jorge Lora, José I. Hormaza

**Affiliations:** Instituto de Hortofruticultura Subtropical y Mediterránea La Mayora (IHSM-UMA-CSIC), Málaga, Spain

**Keywords:** *Annona*, Annonaceae, karyotype, polyploidy, triploid, tetraploid, unreduced gametes

## Abstract

Genome duplication or polyploidy is one of the main factors of speciation in plants. It is especially frequent in hybrids and very valuable in many crops. The genus *Annona* belongs to the Annonaceae, a family that includes several fruit tree crops, such as cherimoya (*Annona cherimola*), sugar apple (*Annona squamosa*), their hybrid atemoya (*A. cherimola* × *A. squamosa*) or pawpaw (*Asimina triloba*). In this work, genome content was evaluated in several *Annona* species, *A. triloba* and atemoya. Surprisingly, while the hybrid atemoya has been reported as diploid, flow cytometry analysis of a progeny obtained from an interspecific cross between *A. cherimola* and *A. squamosa* showed an unusual ploidy variability that was also confirmed karyotype analysis. While the progeny from intraspecific crosses of *A. cherimola* showed polyploid genotypes that ranged from 2.5 to 33%, the hybrid atemoyas from the interspecific cross showed 35% of triploids from a total of 186 genotypes analyzed. With the aim of understanding the possible implications of the production of non-reduced gametes, pollen performance, pollen size and frequency distribution of pollen grains was quantified in the progeny of this cross and the parents. A large polymorphism in pollen grain size was found within the interspecific progeny with higher production of unreduced pollen in triploids (38%) than in diploids (29%). Moreover, using PCR amplification of selected microsatellite loci, while 13.7% of the pollen grains from the diploids showed two alleles, 41.28% of the grains from the triploids amplified two alleles and 5.63% showed up to three alleles. This suggests that the larger pollen grains could correspond to diploid and, in a lower frequency, to triploid pollen. Pollen performance was also affected with lower pollen germination in the hybrid triploids than in both diploid parents. The results confirm a higher percentage of polyploids in the interspecific cross, affecting pollen grain size and pollen performance. The occurrence of unreduced gametes in *A. cherimola*, *A. squamosa* and their interspecific progeny that may result in abnormalities of ploidy such as the triploids and tetraploids observed in this study, opens an interesting opportunity to study polyploidy in Annonaceae.

## Introduction

Polyploidy is believed to be a major mechanism of adaptation and speciation, recognized as a major force in evolution (Van de Peer et al., 2017) and very valuable for crop improvement (Udall and Wendel, 2006; Mason, 2016). Polyploidy is more common in plants than in animals. It is estimated that between 30 and 70% of extant flowering plant species are polyploids (Bretagnolle and Thompson, 1995; Ramsey and Schemske, 1998; Otto and Whitton, 2000; Adams and Wendel, 2005) and all angiosperms are supposed to have descended from polyploidy ancestors and, consequently, would indeed be paleopolyploids (Debodt et al., 2005; Cui et al., 2006; Jaillon et al., 2007; Soltis et al., 2009; Amborella Genome Project, 2013).

Diploid species have two sets of homologous chromosomes. Each chromosome of one set may pair with a corresponding one of the other set during meiosis. Such normal meiosis produces haploid gametes. Abnormal meiosis, due to various genetic and environmental factors, can generate unreduced gametes, or gametes with the somatic chromosome number (Ahmad et al., 1984; Saini, 1997; Viccini and De Carvalho, 2002; Sun et al., 2004; Bajpai and Singh, 2006; Rezaei et al., 2010; Wang et al., 2017). Studies on polyploid evolution have revealed that the most common mechanism of polyploidy in flowering plants involve unreduced gametes (Bretagnolle and Thompson; Darlington, 1937, 1965; Harlan and deWet, 1975; deWet, 1979; Karpechenko, 2010; Kreiner et al., 2017). Different theoretical models of polyploidy have been elaborated considering that the success of tetraploids arisen by sexual polyploidization within a diploid population is influenced by the frequency with which unreduced gametes are produced by diploids (Felber, 1991; Felber and Bever, 1997; Ramsey and Schemske, 1998; Li et al., 2004). There are two main ways to produce unreduced diploid gametes in plants, as a result of first-division restitution (FDR) or second-division restitution (SDR) during meiosis (Hermsen, 1984; De Storme and Geelen, 2013). In FDR, the first meiotic division fails and, as consequence, the two chromosomes of the unreduced gamete are non-sister chromatids. In SDR, the second meiotic division fails and the two chromosomes of the unreduced gamete are sister chromatids. A third proposed mechanism has been reported as indeterminate meiotic restitution (IMR), which produces microspores with disproportionate number of chromosomes due to a restitution mechanism (Lim et al., 2001; De Storme and Geelen, 2013).

The potential role of unreduced gametes in the origin and evolution of polyploids as well as in plant breeding, has been reviewed in different plant species (Harlan and deWet, 1975; deWet, 1979; Bretagnolle and Thompson, 1995; Ramsey and Schemske, 1998; Brownfield and Kohler, 2011; Sattler et al., 2016). The use of unreduced gametes in plant breeding is an effective tool for the induction of polyploidy and variation (Bringhurst and Köhler, 1984; Negri and Veronesi, 1989; Iwanaga et al., 1991; Dewitte et al., 2009, 2010; Younis et al., 2014; Bradshaw, 2016).

The Annonaceae is the largest extant family in the early-divergent eumagnoliid angiosperm clade (The Angiosperm Phylogeny Group et al., 2016). The Annonaceae contains over 2400 species in more than 130 genera, grouped in four subfamilies (Anaxagoreoideae, Ambavioideae, Annonoideae, and Malmeoideae) and 14 tribes (Chatrou et al., 2012), with a pantropical distribution (Couvreur et al., 2011; Chatrou et al., 2012; Guo X. et al., 2017). A limited number of species in the Annonaceae, belonging to just two genera in the tribe Annoneae of the subfamily Annonoideae [*Annona* L. and *Asimina* Adans., since *Rollinia* A. St.-Hil. has been included in *Annona* (Rainer, 2007)], produce edible fruits. Examples include cherimoya (*Annona cherimola* Mill.), sugar apple (*A. squamosa* L.), soursop (*A*. *muricata* L.), atemoya (a hybrid between *A. cherimola* and *A. squamosa*), custard apple (*A. reticulata* L.), ilama (*A. macroprophyllata* Donn. Sm.), pond apple (*A. glabra* L.), or pawpaw (*Asimina triloba*) (Larranaga et al., 2019). Among them, cherimoya and sugar apple have been used as food source by pre-Colombian cultures in Central and South America (Popenoe, 1989). Their cultivation has continued up to the present day, with a clear niche of expansion in countries with subtropical climates. Pawpaw is a particularly interesting fruit crop in the family because, although the fruit shows an exotic tropical flavor, *Asimina* is the only genus of the Annonaceae adapted to cold climates (Pomper and Layne, 2010; Losada et al., 2017). In spite of the phylogenetic position among early-divergent angiosperms and promising interest as commercial crops, few studies have evaluated ploidy in the tribe Annoneae and several discrepancies have been shown among different works, mainly in the karyotype due to the presence of grouped chromosomes (Kumar and Ranadive, 1941; Asana and Adiata, 1945; Bowden, 1945; Darlington and Ammal, 1945; Bowden, 1948; Miege, 1960; Thakur and Singh, 1965, 1969; Walker, 1972; Morawetz, 1986a,b, 1988; Datta and De, 1990; Folorunso and Olorode, 2008). Most *Annona* species are diploid with the exception of *A. glabra* (Bowden, 1945, 1948; Darlington and Ammal, 1945; Miege, 1960; Thakur and Singh, 1969; Morawetz, 1986a) and *A. lutescens* (Morawetz, 1986a) that have been reported as tetraploid, and the previously named *Rollinia* genus (now included in *Annona*, Rainer, 2007), showing tetraploid (*A. neolaurifolia* and *A. exsucca*, Morawetz, 1986b) and hexaploid species (*A. mucosa* and *A. pulchrinervis*, Walker, 1972; Morawetz, 1986a,b). Triploid mutants have also been occasionally reported in *Asimina triloba* (Bowden, 1949). Interestingly, in the frame of a breeding program in *Annona* an unexpected high proportion of triploid genotypes was found in the progeny of an interspecific cross involving the diploid species, *A. cherimola* and *A. squamosa* (Martin, 2013). Spontaneous triploids from crosses involving diploid parents have been reported in intra- and inter-specific crosses in several genera such as *Citrus* (Esen and Soost, 1971), *Populus* (Bradshaw and Stettler, 1993), *Anthoxanthum* (Bretagnolle, 2001), *Rhododendron* (Ureshino and Miyajima, 2002), *Rosa* (Crespel et al., 2006), and *Asparagus* (Ozaki et al., 2014).

Although polyploidy is one of the main processes involved in angiosperm evolution, the mechanisms behind it have not been evaluated previously in the tribe Annoneae in which ploidy has been reported in several species. After the finding of an unexpected high number of triploids in the progeny of an interspecific *A. cherimola* × *A. squamosa* cross, we decided to carry out a thorough study of ploidy levels in several species of the Annoneae and analyze with detail the reasons behind the production of non-reduced gametes. For that, we first studied ploidy in several *Annona* species (*A. senegalensis*, *A. montana*, *A. glabra*, *A. muricata*, *A. emarginata*, *A. neosalicifolia*, *A. cherimola*, and *A. squamosa*) including the hybrid atemoya (*A. cherimola* × *A. squamosa*) and *Asimina triloba*. Then, we analyzed the ploidy in some backcrosses (*A. cherimola* × atemoya), self-crosses (atemoya × atemoya) and intraspecific crosses (*A. cherimola* × *A. cherimola*). Finally, we evaluated the implications of unreduced gametes on pollen performance, pollen size and frequency distribution of pollen grains in the parents and the progeny that result in polyploidy.

## Materials and Methods

### Plant Material

Adult trees of eight *Annona* species (*A. senegalensis*, *A. montana*, *A. glabra*, *A. muricata*, *A. emarginata*, *A. neosalicifolia*, *A. cherimola* and *A. squamosa*) including the hybrid atemoya (*A. cherimola* × *A. squamosa*) were used in these experiments. We also included *Asimina triloba* because *Asimina* is the only genus in the tribe Annoneae in which the presence of natural triploids has been shown (Bowden, 1949). All the plant material analyzed is located in an *ex situ* germplasm field collection at the IHSM La Mayora-CSIC-UMA, Málaga, Spain, at latitude 36°45′N, longitude 4°4′ W and altitude 35 m. The progeny obtained from an interspecific cross between diploid *A. cherimola* (cv. Fino de Jete, “Fj”) and *A. squamosa* (cv. Thai seedless, “Ts”), four backcrosses (*A. cherimola* × atemoya), two self-crosses (atemoya × atemoya) and seven intraspecific crosses (*A. cherimola* × *A. cherimola*) were also analyzed (Tables 1, 2).

**Table 1:** DNA ploidy levels of *Annona* spp. and *Asimina triloba.*

N° accessions	Species	DNA content of 1C (pg)	Genome size (Mpb)	Ploidy
338	*Annona cherimola*	1.7	1666	2×
1	*Annona squamosa*	1.63	1597	2×
5	*A. cherimola* × *A. squamosa*	1.75	1715	2×
1	*Annona senegalensis*	1.7	1666	2×
1	*Annona muricata*	1.7	1666	2×
1	*Annona emarginata*	1.68	1646	2×
1	*Asimina triloba*	1.7	1666	2×
1	*Annona neosalicifolia*	4.82	4724	6×

**Table 2:** DNA ploidy levels of the progeny from interspecific and intraspecific crosses, backcrosses and self-crosses.

Type of cross	Cross (♀ × ♂)	Diploid	Triploid	Tetraploid	*n*
**Interspecific**					
*A. cherimola* × *A. squamosa*	‘Fj’^2^ × ‘Ts’^2^	121 (65%)	65 (35%)	0 (0%)	186
Backcross (F2)	‘Fj’^2^ × ‘FT9’^3^	3 (100%)	0 (0%)	0 (0%)	3
*A. cherimola* × atemoya	‘Fj’^2^ × ‘FT175’^3^	6 (86%)	1 (14%)	0 (0%)	7
	‘Fj’^2^ × ‘FT197’^3^	17 (89.5%)	1 (5.25%)	1 (5.25%)	19
			x̄ = 3.4%		
**Backcross (F3)**					
*A. cherimola* × atemoya	‘Campas’^2^ × ‘FT16’^4^	0 (0%)	9 (56.25%)	7 (43.75%)	16
Self-cross	‘FT7’^2^ × ‘FT7’^2^	34 (100%)	0 (0%)	0 (0%)	34
Atemoya × atemoya	‘FT20’^2^ × ‘FT20’^2^	59 (99%)	1 (0.6%)	0 (0%)	60
			x̄ = 1%		
**Intraspecific**	‘Bonita’^2^ × ‘Fj’^2^	157 (97.5%)	4 (2.5%)	0 (0%)	161
*A. cherimola* × *A. cherimola*	‘Fj’^2^ × ‘Bonita’^2^	71 (96%)	3 (4%)	0 (0%)	76
	‘SM35’^2^ × ‘Fj’^2^	2 (67%)	1 (33%)	0 (0%)	3
	‘Fj’^2^ × ‘SM35’^2^	4 (80%)	1 (20%)	0 (0%)	5
	‘SP12’^2^ × ‘Pazicas’^2^	82 (92.1%)	7 (7.9%)	0 (0%)	89
	‘SP12’^2^ × ‘León’^2^	84 (81.2%)	16 (18.8%)	0 (0%)	85
	‘León’^2^ × ‘IX-24’^2^	84 (96.6%)	3 (3.4%)	0 (0%)	88
			x̄ = 6.9%		

*Ploidy in the parents: diploids^2^; triploids^3^; tetraploids^4^. Fj, Fino de Jete.*

### Measure of the Relative DNA Content Using Flow Cytometry

Flow cytometry analysis was done with the Cystain UV Precise T Kit (Sysmex, Norderstedt, Germany). Crude nuclei were extracted from young leaves using a sharp razor blade and deposited in a nuclei isolation buffer (Sysmex, Norderstedt, Germany). After chopping, the crude solution was passed through a 30 μm nylon filter and mixed with staining buffer (Sysmex) in a proportion of 1 volume of crude solution to 4 volumes of staining buffer. Relative DNA content was measured by using a Cyflow^®^ PA, Partec. DNA content was quantified relative to 2C DNA content from the tomato cultivar, Moneymaker (1.9 pg) (Bennett et al., 2000). The 2C value corresponds to the DNA content of a somatic diploid nucleus (Doležel et al., 1989; De Rocher et al., 1990). DNA content was based on 3000–5000 nuclei per sample and two independent replicates and was calculated using the following formula (Dolezel and Bartos, 2005): Sample 2C DNA content = [(sample G_1_ peak mean)/(standard G_1_ peak mean)] × standard 2C DNA content (pg DNA). DNA content (pg) was converted to mega base pairs (Mpb), 1 pg = 980 Mpb (Bennett et al., 2000).

### Karyotype

Chromosomes were observed both in flower buds and in young leaves. Flower buds were fixed and stored in 3:1 (v/v) ethanol: acetic acid. The karyotype was evaluated in somatic cells from young pistils that were hydrolyzed using 1N HCl, incubated 30 s at 65°C, and squashed in 1% aceto-carmine under a coverslip. Young leaves were hydrolyzed using 45% acetic acid, heated by flame 5 s and squashed. Then, the slide was introduced in liquid nitrogen for several seconds and stained with a solution of 1 μg/mL of 4′,6-diamidino-2-phenylindole (DAPI). The methods were modified from Burley (1965), Tanaka and Okada (1972), and Leeton and Fripp (1991).

### DNA Analysis From Single Pollen Grains

#### Sample Handling

*Annona cherimola* and *A. squamosa* flowering cycles are characterized by a protogynous dichogamous system (Wester, 1910), a common characteristic in Annonaceae (Gottsberger, 1999). Dehiscent anthers from flowers at the male stage of diploid and triploid hybrids and from the two parents of the interspecific cross were shaken using forceps inside 1.5 ml Eppendorf tubes to release the pollen grains; then the pollen grains were stored in a freezer before manipulation. Pollen grains were mixed with 30 μl sterile distilled water in the 1.5 ml Eppendorf tubes and carefully pipetted back and forth. A drop from the mixture of diluted pollen was placed in a disposable Petri dish and observed under a Leica S6D stereo microscope (2.5 × magnification). Some characteristics of the pollen grain such as the rigid and thick exine (Santos and Mariath, 1999; Parre and Geitmann, 2005) and the size from 0.005 to 0.25 mm in diameter (Perveen and Qaiser, 2001; Sarkissian and Harder, 2001) facilitate handling and, as a consequence, the selection of individual pollen grains. However, in *A. cherimola*, as in other Annonaceae (Lora et al., 2009, 2014), the four sibling haploid microspores are held together in a persistent pollen mother cell wall that is surrounded by callose until its dissolution when the microspores are shed free. Because of that, the individual pollen grains had to be mechanically separated by moving them within the drop of water with the help of a single hair paintbrush to reduce static electricity.

#### DNA Extraction

Flow cytometry and chromosome counting do not allow the determination of the parental origin of the extra haploid genome in the triploids. However, polymorphic, heterozygous and codominant markers, like microsatellites, provide an interesting tool to analyze the ploidy level and the genetic origin. This approach can even be used with single pollen grains. For the genotyping analyses, pollen grains were collected from flowers just before anther dehiscence in 15 diploid and 15 triploid hybrids from the same interspecific cross. Twenty pollen grains were isolated per genotype. After collection, individual pollen grains were transferred to a DNA-free PCR tube (0.2 ml capacity) containing 2 μl of extraction buffer (Isagi and Suyama, 2011): 0.01% sodium dodecyl sulfate, SDS, 0.1 g/l proteinase K, 0.01 M Tris-HCl pH 7.8 and 0.01 M EDTA. Following this step, the presence of a single pollen grain in the drop of buffer was checked under the stereo microscope. The DNA extract from the single pollen grain was used directly as a PCR template. The PCR tube was then closed and incubated at 37°C for 60 min and 95°C for 11 min.

#### Single Pollen Grain Genotyping

The multiplex PCR method (Chamberlain et al., 1988) was used to amplify multiple microsatellite loci simultaneously in a single reaction. Genotypes of each pollen grain were analyzed using two microsatellite primers developed in *A. squamosa* LMTS52 and LMTS135 (GenBank KF010995 and KF011078, respectively). Multiplex PCR amplification was performed using a thermal-cycler (Bio-Rad Laboratories, Hercules, CA, United States) under the following conditions: an initial step of activation at 94°C for 1 min, then 35 cycles of denaturation at 94°C for 30 s, annealing at 50°C for 30 s, and extension at 72°C for 1 min, followed by a final extension at 72°C for 5 min. The volume of the reaction mixture was 10 μl containing extracted DNA from a single pollen grain, 16 mM of (NH_4_)SO_4_, 67 mM of Tris-HCL, pH 8.8, 0.01% of Tween20, 4 mM of MgCl_2_, 10 mM de KCl, 0.1 mM of each dNTP, 2.6 μM of each primer and 0.9 U de BioTaq^TM^ DNA polimerase (Bioline). Forward primers were labeled with a fluorescent dye on the 5′ end. PCR products were analyzed by capillary electrophoresis in a CEQ^TM^ 8000 genetic analyzer (Beckman Coulter, Fullerton, CA, United States).

### Occurrence and Frequency of *2n* Pollen Grains

The identification and analysis of the frequency of unreduced microspores from diploid and triploid plants were also made by analyzing the distribution of pollen grain size. To measure pollen size, 0.05 g of anthers from two flowers in the male stage per genotype were placed over a microscope slide. After adding a drop of 45% acetic acid, the anthers were pressed with forceps to release the pollen grains and then the remnant of the anthers removed. Autofluorescence from the exine of the pollen grains allowed visualizing the samples without staining. Preparations were observed under an epifluorescent Leica DM LB2 microscope with a 340–380 excitation filter. Measurements of the diameters of pollen grains in both parents and the hybrids were made with the Image J software over pictures taken with a Canon Power Shot S50 camera attached to the microscope.

The diameter of a minimum of 200 pollen grains per flower was measured and the range and distribution of pollen size was calculated for each plant. The frequency of *2n* pollen grains was recorded by counting all the pollen grains of each sample and separating them into ranges of five units, according to their size. Following the distribution frequency of the pollen diameter, a threshold that corresponded to the mean size in each individual, above which the pollen grains were considered as unreduced, was established. The percentage of unreduced microspores was calculated as the accumulative frequency above the mean size diameter. Some pollen grains with irregular shapes were observed in the samples; those irregular grains, together with those poorly developed, were not considered to determine the pollen diameter. A Student’s *t*-test at the 0.05 significance level was used to compare the unreduced pollen production between diploid and triploid genotypes. To detect a putative relationship between the unreduced pollen production and ploidy level, the Pearson’s correlation coefficient at the 0.01 significance level was computed. Statistical analyses were performed using SPSS 17.0 statistical software (SPSS Inc., Chicago, IL, United States).

### Pollen Germination

To evaluate *in vitro* pollen germination, pollen was maintained within the dehisced anthers as reported previously (Rosell et al., 1999); the anthers were hydrated leaving them in a glass vial placed in a covered tray with wet filter paper for 60 min at room temperature. Then, approximately 0.02 g of pollen with anthers was placed on a 35 mm Petri dish with 1–2 ml of liquid germination medium at room temperature (Lora et al., 2006, 2012). Pollen was considered as germinated when the length of the tube was longer than the grain diameter. Data were collected from four Petri dishes with at least 200 pollen grains counted in each one. The pollen germination medium consisted of 8% sucrose, 200 mg/l MgSO_4_7H_2_O, 250 mg/l Ca(NO_3_)_2_4H_2_O, 100 mg/l KNO_3_, and 100 mg/l H_3_BO_3_ (Lora et al., 2006).

To evaluate *in vivo* pollen germination, pollen tube growth was documented using squash preparations of stigmas from hand pollinated flowers kept in water at room temperature. For this purpose, pistils were fixed in formalin-acetic acid-alcohol (FAA) 24 h after pollination and stored at room temperature. Pistils were water washed and placed in 1N NaOH for 1 h to soften the tissues. Stigmas were dissected and squash preparations were stained with 0.1% aniline blue in PO_4_ K_3_ (Currier, 1957; Linskens and Esser, 1957) and observed with a 340–380 nm excitation filter and an LP425 barrier filter.

## Results

### Ploidy in *Annona*

To study polyploidy in the tribe Annonae, we first analyzed the ploidy and DNA content in eight *Annona* species and *Asimina triloba* (Table 1). *Annona cherimola*, *A. squamosa*, *A. senegalensis*, *A. muricata*, *A. emarginata*, and *Asimina triloba* showed a DNA content between 1.63 and 1.75 pg that correspond to ploidy level 2×. To the best of our knowledge, this is the first reported ploidy of *A. senegalensis*, showing diploidy, and *A. neosalicifolia*, which showed a higher DNA content of 4.82 pg and, consequently, can be considered as hexaploid (Table 1).

**FIGURE 1:**
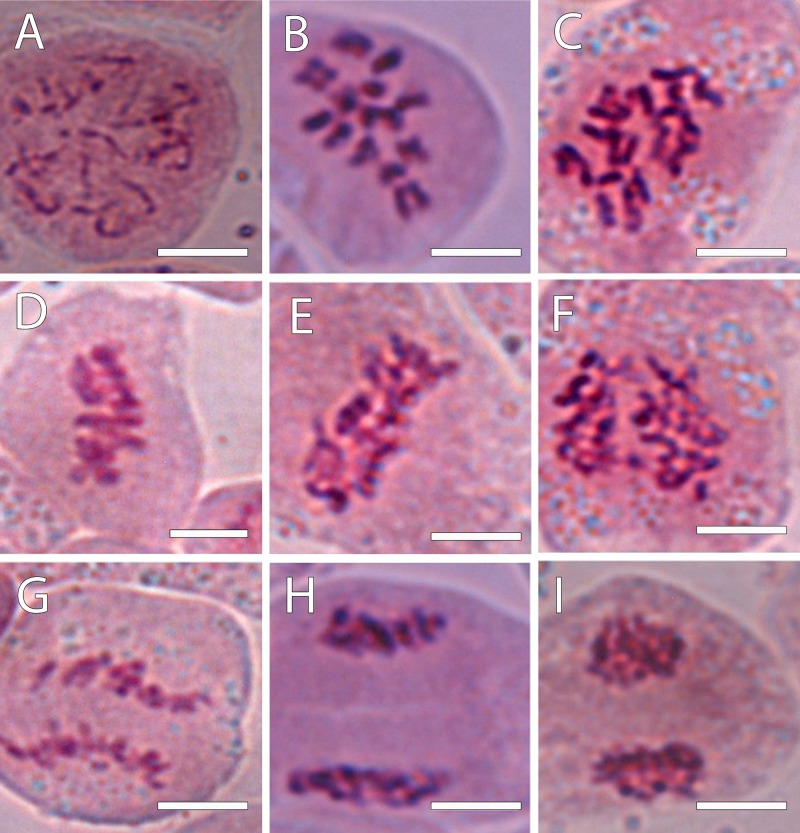
Chromosomes revealed using 1% acetocarmine from the progeny of the interspecific cross *A. cherimola* × *A. squamosa* during the mitotic cycle. **(A)** Chromosomes in a diploid hybrid during early prophase. **(B,C)** Prometaphase in a diploid **(B)** and triploid hybrid **(C)**. **(D,E)** Metaphase in a diploid **(D)** and triploid hybrid **(E)**. **(F)** Chromosomes in a triploid hybrid in an early anaphase. **(G)** Late anaphase in a diploid hybrid. **(H,I)** Telophase in a diploid hybrid. Scale bars, 10 μm.

**FIGURE 2:**
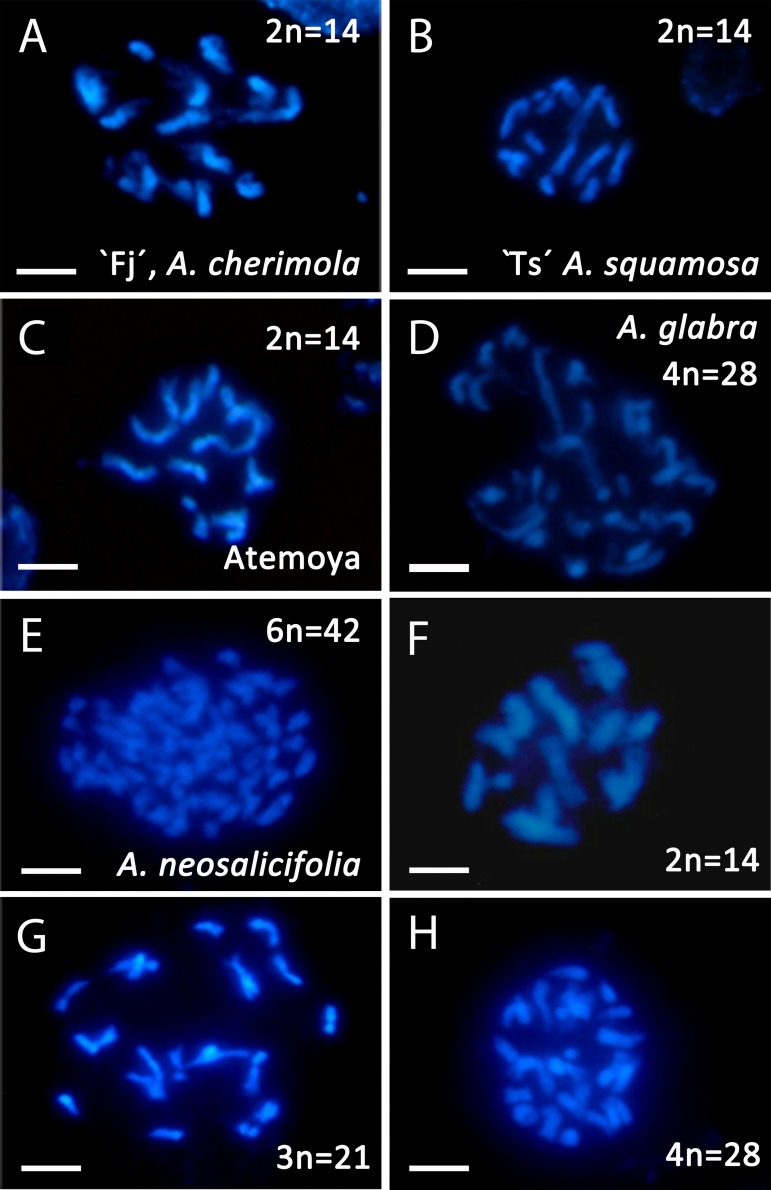
Somatic chromosomes revealed using DAPI staining in *Annona* spp. **(A)** Diploid *A. cherimola*, cv. Fino de Jete, “Fj,” **(B)** Diploid *A. squamosa*, Thai seedless “Ts,” **(C)** Diploid atemoya. **(D)** Tetraploid *A. glabra*. **(E)** Hexaploid *A. neosalicifolia*. **(F,H)** Diploid hybrid **(F)**, triploid hybrid **(G)** and tetraploid hybrid **(H)** from the interspecific cross *A. cherimola* × *A. squamosa*. Scale bars, 10 μm.

To confirm the ploidy in the *Annona* species studied, we evaluated the karyotype using acetocarmine (Figure 1) and DAPI (Figure 2). The acetocarmine staining in somatic cells from young flower buds revealed all different phases of mitosis (Figure 1). More specifically, DAPI staining revealed the number of somatic chromosomes that confirmed the abovementioned ploidy. Thus, *A. cherimola*, *A. squamosa*, the diploid atemoyas observed by flow cytometry analysis and *A. muricata* showed 14 somatic chromosomes (Figure 2). Additionally, we also quantified the number of chromosomes of *A. glabra* that was 28 (Figure 2D) confirming its 4× ploidy level (Bowden, 1948). The hexaploidy of *A. neosalicifolia* was also confirmed by its karyotype showing 42 chromosomes (Figure 2E). Thus, the karyotype and the flow cytometry analyses revealed that the basic chromosome number in *Annona* is seven.

### Polyploidy in the Interspecific Cross *A. cherimola × A. squamosa*

*Annona cherimola* and *A. squamosa* can be intercrossed to produce the cultivated *A. squamosa* × *A. cherimola* hybrid, atemoya. We analyzed DNA content in five accessions of atemoya and the results showed a ploidy of 2× (Table 1). To further examine ploidy in atemoya, we also studied the ploidy in 186 atemoyas of the progeny from the interspecific cross *A. cherimola* (cv. Fino de Jete, “Fj”) × *A. squamosa* (Thai seedless. “Ts”). Surprisingly, the interspecific cross showed 65 triploid individuals (35%, Table 2). Thus, additionally, we also studied the progeny from four backcrosses (*A. cherimola* × atemoya), two self-crosses (atemoya × atemoya) and seven intraspecific crosses (*A. cherimola* × *A. cherimola*). The resulting progeny showed diploid, triploid and, interestingly, tetraploid individuals (Table 2). Three of the backcrosses were performed using pollen from triploid atemoyas and triploid individuals were observed in the progeny of two of them. The fourth backcross was performed in the two directions with the tetraploid individual. No fruits were obtained when the tetraploid genotype was used as maternal parent but fruits were obtained when it was used as male parent, showing 56% of triploids and 44% of tetraploids in the progeny. Anomalies in ploidy were also frequent in the intraspecific crosses showing 6.9% of triploid individuals and was less frequent in the self-crosses (Table 2). Consistent with the flow cytometry data, the karyotype of diploid, triploid and tetraploid atemoyas from the interspecific cross *A. cherimola* × *A. squamosa* showed 21 and 28 chromosomes, respectively (Figure 2F–H). Taken together, anomalous ploidy was more frequent in interspecific crosses than in intraspecific crosses.

### DNA Amplification and Genotyping From Single Pollen Grains

Genotyping of 20 pollen grains per genotype was performed with two microsatellite markers, LMTS52 and LMTS135, which amplified two alleles in the female parent, *A. cherimola* “Fj” and only one in the male parent, *A. squamosa* “Ts.”

Within each genotype, we observed pollen grains with patterns of only one peak, diallelic and triallelic patterns with different frequencies in triploids. Among the diploid interspecific hybrids analyzed, 13.7% of the pollen grains showed two alleles for the same locus. Among interspecific triploid hybrids, 41.28% of the pollen grains had two alleles and 5.63% three peaks corresponding to three alleles with clear and stable amplification signals.

### Pollen Performance Was Affected in Triploid Hybrids

**FIGURE 3:**
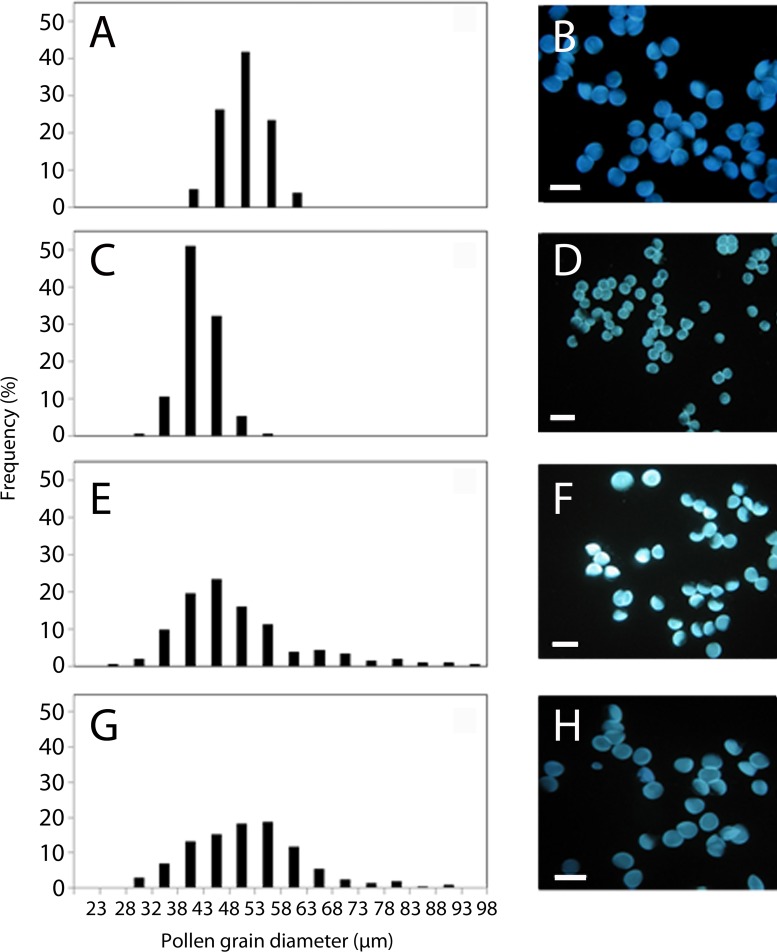
Frequency distribution and images showing the heterogeneity of pollen diameters in the parental genotypes, *Annona cherimola* cv. Fino de Jete, “Fj” **(A,B)** and *Annona squamosa*, Thai seedless “Ts” **(C,D)**, in the FT148 hybrid diploid **(E,F)** and in the FT6 hybrid triploid **(G,H)** from the interspecific cross between *A. cherimola* × *A. squamosa*. Scale bars, 10 μm.

Changes of nuclear DNA content could affect pollen performance. Thus, we next studied pollen grain size and pollen germination. Differences in size among pollen grains from the same genotype were observed in the samples analyzed in this study (Figure 3).

A high polymorphism in the size of pollen grains was detected within the interspecific progeny. Among the analyzed diploid hybrids, pollen grains with sizes ranging from 22 to 100 μm were observed with an average pollen size of 52 ± 10 μm. In the case of the triploid hybrids, pollen grain size ranged from 18 to 126 μm with an average size of 56 ± 11 μm. Regarding the closely related parental genotypes, pollen grain size ranged from 43 to 63 μm with an average of 52 ± 4 μm in the female parent (*A. cherimola*, “Fj”), and from 33 to 58 μm with an average of 44 ± 4 μm in the male parent (*A. squamosa*, “Ts”).

We established a threshold in pollen grain size in each analyzed individual corresponding to its mean size to determine the percentage of unreduced pollen. Thus, in general, a higher proportion of unreduced pollen grains was observed in triploids (38 ± 9%) than in diploids (29 ± 10%). Moreover, we observed “giant” grains both in diploids and triploids with diameters larger than 75 μm. The frequency distribution of pollen size of parents and diploid and triploid progeny was clearly distinct (Figure 3). While in the parental genotypes mainly three pollen grain diameters can be distinguished (Figure 3A–D), both diploid and triploid hybrids showed an overlap in size distribution between reduced and unreduced pollen and a larger variation in the frequency distribution of pollen grain size (Figure 3E–H). The production of unreduced pollen differed statistically between diploids and triploids, (*t* = −3.01, df = 38, *P* = 0.005). The distribution was unimodal and symmetrical in diploids whereas it was skewed toward larger diameter values in triploids (Figure 3). A significant correlation was observed between the frequency of unreduced pollen and the ploidy level of the hybrids (Pearson’s correlation coefficient *r* = 0.439, *P* = 0.005, *N* = 40). Additionally, individual frequency distribution data revealed that some genotypes from the interspecific progeny, including diploids and triploids, are more capable of producing unreduced pollen than others.

**FIGURE 4:**
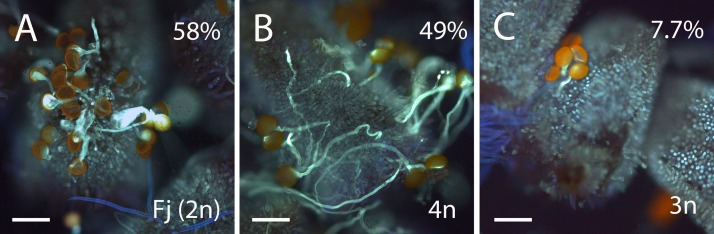
Aniline-blue-stained squash mounts of stigmas of *Annona cherimola* showing germination of pollen from diploid *A. cherimola*, cv. Fino de Jete “Fj” **(A)**, tetraploid **(B),** and triploid **(C)** hybrid. Scale bars, 100 μm.

We next evaluated if these morphological differences among pollen grains result in differences in pollen germination. Percentage of pollen germination *in vitro* in triploid hybrids ranged from 15 to 45% with an average of 27%. By contrast, pollen germination *in vitro* in diploid genotypes ranged between 21 and 72% with an average of 36%. Pollen germination *in vitro* was higher in the parental lines (75% in “Fj” and 55% in “Ts”) compared with triploid (19%) and diploid (36%) hybrids. We evaluated pollen germination *in vivo* on the stigma using pollen from the parental line, “Fj”, a triploid hybrid and a tetraploid hybrid from the backcross “Fj” × “FT197.” Pollen from “Fj” showed higher germination (58% ± 3.8, *n* = 646) than pollen from a triploid hybrid (7.7% ± 0.4, *n* = 267). Pollen from a tetraploid hybrid also showed high germination (49% ± 10.5, *n* = 419, Figure 4).

## Discussion

Polyploidization is one of the main force of evolution and crop improvement in flowering plants. Although most species of the genus *Annona* show diploidy, the progeny of a cross between diploid *A. cherimola* and *A. squamosa* showed an unexpected high polyploidy level with a high number of triploid hybrids. Consequently, in order to understand the reasons of this unexpected high number of triploids, we analyze the polyploidy and its consequences for pollen performance in different species of the genus *Annona* and in *Asimina triloba*.

### Polyploidy in Annonaceae

It has been suggested that the ancestral basic chromosome number in Annonaceae is seven (Walker, 1972), that has also been considered as the basic chromosome number in flowering plants (Grant, 1963). However, in the tribe Annoneae, a haploid chromosome number of seven has only been reported in the genus *Annona*; in this tribe, *x* = 8 has been reported in the genus *Asimina* (Tanaka and Okada, 1972), *Disepalum* (Johnson, 1989), *Goniothalamus* (Sauer and Ehrendorfer, 1984), and *Neostenanthera* (Walker, 1972). Moreover, *x* = 8 has also been reported in sister lineages such as the tribes Monodoreae (genera *Isolona*, *Monodora*, and *Uvariopsis*) (Walker, 1972) and Uvarieae (genera *Dasymaschalon*, *Desmos*, *Fissistigma*, *Friesodielsia*, *Melodorum*, *Mitrella*, *Sphaerocoryne*, and *Uvaria*) (Walker, 1972; Okada and Ueda, 1984; Sauer and Ehrendorfer, 1984; Van Heusden, 1992) and in the more basal tribes Duguetieae (genera *Duguetia* and *Fusaea*) and Xylopieae (genera *Artabotrys* and *Xylopia*) (Morawetz, 1984; Van Heusden, 1992). This could indicate that *x* = 7 in *Annona* may have arisen by aneuploid chromosome loss from ancestors with *x* = 8. Indeed, Morawetz (1986a) already proposed *x* = 8 as the original basic chromosome number in Annonaceae. In this work we have observed the haploid chromosome number seven in *A. cherimola*, *A. squamosa* and their hybrid, atemoya, which supports previous karyotype reports in both species (Kumar and Ranadive, 1941; Asana and Adiata, 1945; Thakur and Singh, 1965, 1969; Tanaka and Okada, 1972; Morawetz, 1986a) in contrast to *x* = 8 reported by Bowden (1945, 1948). The presence of the eighth chromosome could be due to the confusion of distant satellite (or whole arms) with separate chromosomes (Sauer and Ehrendorfer, 1984). Polyploidy has been reported in nine genera of the Annonaceae (Van Heusden, 1992), mainly tetraploids although triploids have also been reported in *Cymbopetalum* (Morawetz, 1986a) and *Duguetia* (Morawetz, 1984). Hexaploid and octoploid ploidy levels were also observed in the former genus *Rollinia* (Walker, 1972; Van Heusden, 1992) that is now included in the genus *Annona* (Rainer, 2007). In this work, this hexaploidy was also observed in *Annona neosalicifolia* previously named *Rollinia neosalicifolia.*

Although triploid mutants have been occasionally reported in *Asimina triloba* (Bowden, 1949), their presence is generally unknown in the tribe Annoneae. Interestingly, we observed a high percentage of triploid hybrid atemoyas (35%) from an interspecific cross between *A. cherimola* × *A. squamosa*. However, atemoya has been considered diploid (Bowden, 1948). The triploid hybrids may result from the fusion of reduced (*n*) and unreduced (*2n*) gametes. The production of unreduced pollen grains is mainly controlled genetically (Mok et al., 1975; Ramsey and Schemske, 1998; Barcaccia et al., 2003; De Storme and Geelen, 2011; Mason and Pires, 2015), although there are several evidences, in many crop species, showing that the genes involved in the control of the unreduced pollen production are highly influenced by climatic conditions (Bretagnolle and Thompson, 1995; Ramsey and Schemske, 1998; Bretagnolle, 2001; De Storme et al., 2012). Studies in different species of Solanaceae, Salicaceae, Poaceae, Rosaceae, and Ranunculaceae have shown the influence of the environmental conditions, mainly light and temperature, in the production of *2n* gametes (Veilleux and Lauer, 1981; Hermsen, 1984; Felber, 1991; Fuzinatto et al., 2008; Rezaei et al., 2010; Pecrix et al., 2011; Guo L. et al., 2017). Particularly changes on temperatures prevailing at the gamete formation stage can cause various meiotic abnormalities due to the change in the expression of some genes that affect gamete viability (Kumar and Singhal, 2011; Singhal et al., 2011; Pecrix et al., 2011; Guo L. et al., 2017). A similar situation could be occurring in *Annona* in this work, where crosses were performed in a region with different temperature ranges than those present in the natural range of cultivation in the Neotropics during flowering time. While cherimoya is cultivated under an average annual temperature range of 18–21°C in Ecuador with limited annual fluctuations (Van Damme et al., 2000) or between a temperature range of 18°C and 25°C in the summer in Peru (Morton, 1987), under the growing conditions of Southern Spain, the fluctuation is higher with average high temperatures during the flowering season ranging from 19 to 29°C (Lora et al., 2011). Interestingly, this production of triploids is only observed in the progeny of the crosses made under these environmental conditions, since all the five accessions of atemoyas and the 338 accessions of *A. cherimola* maintained at the IHSM la Mayora *Annona* germplasm collection are diploids.

### DNA Amplification and Genotyping From Single Pollen Grains Revealed the Presence of Unreduced Pollen Grains

Due to the small size of pollen and the difficulty of manipulation, DNA extraction from single pollen grains have been tried by laborious methods such germination of the pollen (Ziegenhagen et al., 1996; Honsho et al., 2016) and drilling into the pollen wall by using a UV-laser microbeam (Matsunaga et al., 1999). However, these methods are unsuitable for processing a large number of samples. In this work we optimized the approach of using single pollen grains through the application of multiplex PCR or co-amplification of several microsatellite markers in the same reaction (Ghislain et al., 2004; Meudt and Clarke, 2007; Guichoux et al., 2011) to amplify the haploid nuclear genome of a single pollen grain. The markers used in this work allowed us detect a high heterozygosity transmission level from the pollen donor plant to the progeny in which FDR seems to be involved. However, heterozygous markers for the male parent would be necessary to infer a possible SDR situation.

Triploids usually produce euploid gametes (*n*, *2n*) (Belling and Blakeslee, 1922; Dermen, 1931; King, 1933; Satina and Blakeslee, 1937a,b; Lange and Wagenvoort, 1973; Dujardin and Hanna, 1988; Kovalsky et al., 2018) but also *3n* gametes through gamete non-reduction (Belling and Blakeslee, 1922; Lange and Wagenvoort, 1973; Mok et al., 1975). Thus, as described previously in other species, among the triploid interspecific hybrids between *A. cherimola* and *A. squamosa* we observed a frequency of 53% of pollen grains with one allele (*n*), 41.3% with two alleles (*2n*) and 5.63% with three alleles (*3n*). It should be pointed out, however, that diploid pollen grains containing two copies of the same allele will be considered as haploids and, consequently, the number of *2n* pollen grains detected is the minimum, and the real number would be probably higher. As expected, a larger percentage of gametes with two alleles was observed in the triploid hybrids that showed approximately three times more unreduced gametes than diploids.

### Pollen Performance Was Affected in Unreduced Pollen

DNA content can have a direct effect on pollen morphology and performance. Indeed, the examination of the size range of the pollen produced by an individual is the most common method to detect unreduced pollen. This method has been used as indicator of *2n* pollen presence in several species (Quinn et al., 1974; Ramanna, 1983; Sala et al., 1989; Orjeda et al., 1990; Bretagnolle, 2001; Crespel et al., 2006; Kovalsky and Solís Neffa, 2012; Nikoloudakis et al., 2018). In these studies, pollen grains with larger size have usually been considered as unreduced.

In this work, pollen grains showed larger diameters in triploid than in diploid genotypes. Size distribution of pollen grains revealed a relative overlap between unreduced and reduced pollen as described previously in other species (Maceira et al., 1993; Yan et al., 1997; Bretagnolle, 2001). Therefore, based on visual observations, we used a pollen grain size threshold with the mean size to determine the unreduced pollen grains produced in the parents and in each diploid and triploid genotype analyzed. A large variation was found in the production of unreduced pollen between triploid (38%) and diploid (29%) genotypes and a high positive correlation between the production of unreduced pollen grains and the ploidy level of the pollen-producing plant has been observed. However, some of the large pollen may not be *2n* (Maceira et al., 1993) and could include *3n* gametes. The frequency of unreduced gametes produced by the diploid interspecific hybrids was higher than that observed in other species such as *Turnera sidoides* (Kovalsky and Solís Neffa, 2012) or *Anthoxanthum alpinum* (Bretagnolle, 2001) with low frequency of unreduced pollen production (1% approximately) but, lower than those found in diploid population of *Populus tomentosa* with up to 50% of unreduced gametes (Zhang and Kang, 2010).

We also observed giant pollen grains with sizes higher than 75 μm both in the diploid and triploid progeny, although it occurred more frequently in triploids. These giant pollen grains may be *3n* pollen (Tavoletti et al., 2000; Camadro et al., 2008; Zhang and Kang, 2010). The production of these *3n* gametes would increase the probability of obtaining tetraploid plants in the progeny obtained after crossing diploid and triploid genotypes.

Our data also revealed that some genotypes from the interspecific progeny seemed prone to produce a higher percentage of unreduced pollen than others. Similar patterns have been observed in other plant species (Watanabe and Peloquin, 1989; Haan et al., 1992; Maceira et al., 1992). The frequency of *2n* gametes was also found variable within the flowers of an individual tree in some cases such as in *Medicago sativa* (McCoy et al., 1982; Veilleux et al., 1982), *Solanum* spp. (Veilleux, 2011), and *Turnera sidoides* (Kovalsky and Solís Neffa, 2012, 2016). The capacity to produce *2n* gametes is governed by different alleles with different degrees of penetrance and expressivity (Bretagnolle and Thompson, 1995), and this could explain the individual variation observed within our population.

In addition to pollen size, pollen viability and germination could be affected by chromatin distribution imbalance in triploids (Bretagnolle and Thompson, 1995; Ramsey and Schemske, 1998). We observed a reduced pollen germination (7.7%) in triploid compared to the diploid (58%) genotypes. Reduction of pollen viability was reported in triploids of *Manihot esculenta* (cassava) (Nassar, 1992) and rose (Crespel et al., 2006) showing reductions of 19 and 50%, respectively. However, in spite of the reduced pollen viability and germination, the evidence of the functionality of the pollen from triploid hybrid atemoyas was observed in the backcrosses. Similarly, functional pollen from triploids was also reported in potato (Mok et al., 1975; Tarn and Hawkes, 1986; Adiwilaga and Brown, 1991), and *Lolium* (Thomas et al., 1988) and, consequently, they could be used for introgression of genetic diversity from diploid to polyploid crop varieties (Bretagnolle and Thompson, 1995).

### Polyploidy, Evolurtion and Crop Improvement

Recently, molecular tools have provided an interesting insight into the regulatory and genomic consequences of polyploidy. Together with the emerging evidence of ancestral duplication through polyploidization in model plants, knowledge of these consequences has stimulated thinking on the relationship between early polyploidy events, success of the polyploids, and the long-term fate of the new species (Comai, 2005). The “*2n* gametes pathway” is considered to be the main way of polyploidy origin and evolution in flowering plants (Harlan and deWet, 1975; Bretagnolle and Thompson, 1995; Ramsey and Schemske, 1998; De Storme and Geelen, 2013). Although unreduced pollen grain production has been observed in several species (Bretagnolle, 2001; Ramanna and Jacobsen, 2003; Taschetto and Pagliarini, 2003; Crespel et al., 2006; Gallo et al., 2007; Camadro et al., 2008; Zhang et al., 2009; Gómez-Rodríguez et al., 2012; Xue et al., 2011), the observations made in this work in two different species (*A. cherimola* and *A. squamosa*) and their interspecific hybrids could have implications to explain the emergence of polyploidy and as valuable information for crop improvement in the Annonaceae. Additional work will be needed to explain the reasons behind the unexpected high production of polyploid genotypes in the interspecific and intraspecific crosses in *Annona*. In any case, polyploidy provides genome buffering, higher allelic diversity and the possibility of new functions for the duplicated genes; all this has important implications for crop improvement (Udall and Wendel, 2006; Mason, 2016) and, indeed, many of the current cropsr are hybrids of polyploids (Mason, 2016). Interestingly, most of the studies of polyploidy in crops have been performed in non-perennial species (Mason, 2016) and, consequently, there is a lack of information on the mechanisms and extension of polyploidy in most woody perennial crops, such as those studied in this work. The approaches shown here to study the reasons behind the unexpected high number of polyploids produced in intraspecific and interspecific crosses involving diploid genotypes can be useful to perform similar analyses in other woody perennial crops.

## Author Contributions

CM, MAV, and JIH conceived the study and designed the experiments. CM performed most of the experiments. JL performed some of the additional crosses, analyzed the progeny and the *in vivo* pollen germination experiments. CM, JL, MAV, and JIH wrote the manuscript.

## Conflict of Interest Statement

CM is currently employed by company Rijk Zwaan Ibérica S.A. The remaining authors declare that the research was conducted in the absence of any commercial or financial relationships that could be construed as a potential conflict of interest.
